# The HARP (Hemoglobin–Albumin–C-Reactive Protein) Index: A Biologically Designed Composite Biomarker for Continuous Prognostic Modeling in Locally Advanced Nasopharyngeal Carcinoma

**DOI:** 10.3390/diseases14090306

**Published:** 2026-08-25

**Authors:** Erkan Topkan, Efsun Somay, Sibel Bascil, Duriye Ozturk, Ugur Selek

**Affiliations:** 1 Department of Radiation Oncology, Faculty of Medicine, Başkent University, Adana 01250, Türkiye; docdretopkan@gmail.com; 2 Department of Oral and Maxillofacial Surgery, Faculty of Dentistry, Başkent University, Ankara 06540, Türkiye; 3 Department of Periodontology, Faculty of Dentistry, Başkent University, Ankara 06540, Türkiye; bascil5@yahoo.com; 4 Department of Radiation Oncology, School of Medicine, Afyonkarahisar Health Sciences University, Afyonkarahisar 03030, Türkiye; duriyeozturk07@gmail.com; 5 Department of Radiation Oncology, School of Medicine, Koç University, Istanbul 34330, Türkiye; ugurselek@yahoo.com

**Keywords:** nasopharyngeal carcinoma, HARP index, prognosis, chemoradiotherapy, survival analysis

## Abstract

Background/Objectives: To evaluate the prognostic significance of the novel hemoglobin–albumin–C-reactive protein (HARP) index in patients with locally advanced nasopharyngeal carcinoma (LANPC) treated with definitive concurrent chemoradiotherapy (CCRT). Methods: This retrospective study included 248 patients with LANPC treated with definitive CCRT between 2011 and 2020. The HARP index [hemoglobin × (albumin ÷ C-reactive protein)] was calculated using pretreatment laboratory values. Prognostic associations between HARP and survival outcomes were evaluated using continuous and categorical Cox regression analyses, restricted cubic spline modeling, receiver operating characteristic analyses, and bootstrap-based internal validation. Results: Lower pretreatment HARP values were independently associated with inferior progression-free survival (PFS) and overall survival (OS). In continuous Cox analyses, increasing HARP values were associated with reduced risks of mortality and disease progression (both *p* < 0.001). ROC analysis identified 3.2 as the exploratory cut-off value for clinical stratification. Patients with HARP ≥ 3.2 (n = 112) had significantly better outcomes than those with HARP < 3.2 (n = 136). Median PFS and OS were not reached in the high-HARP group, whereas they were 47.0 and 72.0 months, respectively, in the low-HARP group. Low HARP values were associated with inferior PFS (HR, 3.86; *p* < 0.001) and OS (HR, 3.06; *p* < 0.001). Bootstrap internal validation demonstrated stable model discrimination with minimal optimism. Conclusions: The novel HARP index is an independently associated and internally validated prognostic biomarker in patients with LANPC treated with definitive CCRT. HARP may provide a practical tool for improved risk stratification, pending prospective external validation.

## 1. Introduction

Given the intricate anatomy of the nasopharynx and its proximity to critical structures, radiotherapy (RT)—particularly intensity-modulated RT (IMRT)—remains the therapeutic cornerstone for non-metastatic nasopharyngeal carcinoma (NPC) across all disease stages [1]. Although RT alone is the preferred modality for early-stage disease [2], the standard of care for locally advanced NPC (LANPC) is platinum-based concurrent chemoradiotherapy (CCRT), delivered with or without induction chemotherapy [3,4,5,6]. Notwithstanding significant advances in RT techniques, chemotherapy administration, toxicity mitigation, and supportive care, the 5-year survival rate for LANPC remains approximately 80% [7]. While locoregional control now exceeds 90%, distant metastasis remains the principal cause of mortality in this cohort [8]. Collectively, these realities underscore the imperative for refined prognostic stratification and personalized therapeutic approaches that incorporate robust tumor- and host-related determinants.

Conventional prognostic evaluation in patients with LANPC is anchored in the tumor–node–metastasis (TNM) classification, with particular emphasis on T- and N-staging. While this anatomical framework provides a standardized means of risk assessment, it is increasingly recognized that clinically salient prognostic determinants extend beyond TNM staging. These include performance status, comorbidity profiles, nutritional indices, systemic inflammatory and immune responses, tumor hypoxia, circulating biomarkers such as lactate dehydrogenase, volumetric tumor burden, Epstein–Barr virus (EBV) DNA titers, extranodal extension, and tumor metabolic activity, as reflected by the maximum standardized uptake value [9]. Despite the American Joint Committee on Cancer (AJCC) TNM system’s pre-eminence in guiding prognostication for NPC [10], its anatomical focus fails to encapsulate the biological heterogeneity and host-related variables that profoundly modulate therapeutic responsiveness and survival [11,12,13]. Thus, patients with ostensibly identical TN stages may experience divergent clinical trajectories despite receiving analogous treatment regimens [14,15]. These considerations highlight an urgent need for more nuanced prognostic models that integrate multidimensional biological and host-related factors to achieve superior risk stratification beyond the scope of conventional anatomical staging.

Nutritional status, immune competence, and systemic inflammatory burden have emerged as pivotal prognostic determinants in patients with LANPC. Among biochemical indices that capture these interrelated physiological domains, serum albumin (Alb) and C-reactive protein (CRP) are especially compelling due to their established clinical utility, universal accessibility, and cost-effectiveness. Within this framework, a growing corpus of evidence underscores the independent prognostic significance of the CRP-to-Alb ratio (CAR) in NPC [16,17,18]. Meta-analytical data further substantiate that elevated pretreatment CAR values are consistently associated with adverse survival outcomes across diverse treatment settings and disease stages [18]. Collectively, these observations affirm CAR as a clinically salient biomarker for refined prognostic stratification in NPC.

Tumor hypoxia constitutes a critical determinant of both prognosis and therapeutic resistance in NPC. Across a spectrum of malignancies—including head and neck cancers—hypoxia is firmly established as a principal mediator of resistance to radiotherapy and chemotherapy, with hypoxic subregions present in more than half of all solid tumors [19,20]. Molecularly, oxygen plays a pivotal role in radiotherapy by stabilizing radiation-induced DNA lesions through interactions with DNA radicals, thereby curtailing subsequent repair mechanisms in irradiated tumor cells [21]. Under hypoxic conditions, diminished oxygen availability impairs this stabilization, facilitating enhanced DNA repair and the persistence of radioresistant cellular subpopulations. Moreover, hypoxia-inducible signaling cascades promote neoangiogenesis, genomic instability, and the evolution of aggressive, apoptosis-resistant tumor phenotypes [22,23]. Within this paradigm, hemoglobin (Hb) serves as an indirect yet clinically accessible surrogate of tumor oxygenation and has demonstrated significant prognostic utility in NPC. Multiple clinical investigations have consistently revealed that anemia or pretreatment Hb concentrations below 11.0 g/dL confer significantly poorer survival outcomes in patients with locally advanced disease undergoing definitive CCRT [14,24].

Although prior investigations have independently demonstrated the prognostic significance of hemoglobin (Hb) and the CAR in LANPC [14,16,17,18,24], integrating these biologically complementary markers into a composite prognostic index has not been previously explored in this clinical context. However, simply combining existing laboratory parameters does not necessarily yield a biologically meaningful composite index. Therefore, HARP was designed according to a predefined biological framework that integrates favorable determinants of oxygen-carrying capacity and nutritional reserve, represented by Hb and Alb, with the adverse influence of systemic inflammation, represented by CRP. To preserve biological interpretability, CAR was intentionally reformulated as the Alb-to-CRP (AC) ratio, ensuring that favorable components (Hb and Alb) contribute positively to the index, whereas the adverse inflammatory component (CRP) contributes inversely. Accordingly, we developed the Hb–Alb–CRP (HARP) index, defined as HARP = Hb × (Alb ÷ CRP). To evaluate this hypothesis, we conducted a retrospective cohort analysis to assess the prognostic significance of HARP using continuous, spline-based, and exploratory categorical survival models in patients with LANPC treated with definitive CCRT.

## 2. Patients and Methods

### 2.1. Study Design and Participants

This retrospective single-center cohort study was conducted at the Department of Radiation Oncology, Baskent University Medical Faculty. Medical records of patients with LANPC who underwent definitive CCRT between June 2011 and December 2020 were reviewed. All eligible patients meeting the predefined inclusion and exclusion criteria were consecutively identified and included. Eligibility criteria included age 18–80 years, Eastern Cooperative Oncology Group (ECOG) performance status 0–2, body mass index (BMI) ≥ 18.5 kg/m^2^, and clinical stage T3–4N0–3M0 or T1–4N1–3M0 disease according to the American Joint Committee on Cancer (AJCC) 8th edition staging system. Additional requirements included histologically confirmed non-keratinizing or undifferentiated squamous cell carcinoma and availability of pretreatment ear–nose–throat (ENT) examination findings, head and neck magnetic resonance imaging (MRI), and fluorodeoxyglucose positron emission tomography–computed tomography (PET-CT). Complete records of radiotherapy, chemotherapy administration, follow-up ENT examinations, and MRI and PET-CT imaging were also required. Because this was a CCRT-based study, patients were additionally required to have completed at least one cycle of platinum-based concurrent chemotherapy during RT.

Patients with prior malignancies, immunosuppressive diseases, previous systemic chemotherapy, or prior RT were excluded. Individuals with confirmed active infections at baseline evaluation or receipt of immunosuppressive medications within 30 days before initiation of CCRT were also excluded to minimize non-tumor-related inflammatory variability. The study was conducted and reported in accordance with the Strengthening the Reporting of Observational Studies in Epidemiology (STROBE) guidelines (Appendix A).

### 2.2. Chemoradiotherapy Protocol

All participants received definitive CCRT using previously reported IMRT and chemotherapy protocols [14]. IMRT was delivered once daily, 5 days per week, over 7 weeks. Concurrent chemotherapy consisted of cisplatin (80 mg/m^2^) administered at 3-week intervals for at least one cycle during RT. Following CCRT, all patients were advised to receive two additional cycles of cisplatin-based doublet chemotherapy if clinically tolerable. Standard supportive care measures, including antiemetic therapy, analgesics, hydration, and nutritional support, were provided according to individual clinical requirements.

### 2.3. Measurement of the HARP Index

The HARP index was calculated using pretreatment Hb, Alb, and CRP values obtained before initiation of CCRT and was defined as follows: HARP = Hb × (Alb/CRP). Hemoglobin was measured in g/dL, serum Alb in g/dL, and CRP in mg/L. Because HARP is unit-dependent, the exploratory cut-off value of 3.2 should be interpreted only when these same units are used. For example, a patient with Hb 12.0 g/dL, Alb 4.0 g/dL, and CRP 15.0 mg/L would have a HARP value of 12.0 × (4.0/15.0) = 3.2.

The HARP index was intentionally constructed to preserve biologically concordant directionality among its components: higher Hb and Alb values, reflecting greater oxygen-carrying capacity and nutritional reserve, respectively, increase the index, whereas higher CRP, reflecting greater systemic inflammatory burden, decreases it. A multiplicative rather than simple additive or subtractive formulation was selected because Hb, Alb, and CRP are measured in different units and on markedly different numerical scales; direct summation of their raw values would therefore assign relative influence partly according to measurement scale rather than biological rationale, whereas an additive model would otherwise require standardization or empirically derived weighting coefficients. The multiplicative structure provides a parsimonious continuous measure in which proportional changes in Hb and Alb contribute positively and proportional changes in CRP contribute inversely, without introducing cohort-specific, outcome-derived weights. Accordingly, the formulation was biologically motivated and prespecified rather than selected through post hoc optimization of survival discrimination in the present dataset. Nevertheless, this rationale does not establish the multiplicative formulation as mathematically superior to alternative standardized, weighted, or transformed approaches, which should be comparatively evaluated together with external validation of HARP in independent cohorts.

### 2.4. Response Assessment

Although the study design was retrospective, treatment response and follow-up evaluations were prospectively recorded in accordance with our institutional surveillance protocol. Patients were assessed every 3 months for the first 2 years after CCRT, every 6 months in years 3–5, and annually thereafter, or more frequently when clinically indicated. At each follow-up visit, an otorhinolaryngology specialist performed an endoscopic examination to evaluate local or regional recurrence and second primary tumors. Positron emission tomography–computed tomography (PET-CT) was used to assess metabolic response and distant metastatic disease until a complete metabolic response was confirmed. Treatment response was evaluated according to the PET Response Criteria for Solid Tumors (PERCIST) [25]. After confirmation of a complete metabolic response, PET-CT surveillance was replaced with head-and-neck magnetic resonance imaging and/or computed tomography. Additional imaging studies were performed only when clinically indicated for suspicious findings or suspected recurrence.

The reporting of this study conforms to the Reporting Recommendations for Tumor Marker Prognostic Studies (REMARK) guidelines [26].

### 2.5. Clinical Endpoints and Statistics

The primary objective of this retrospective cohort analysis was to evaluate the association between the pre-CCRT HARP index and overall survival (OS), defined as the time from initiation of CCRT to death from any cause or last follow-up. The secondary objective was to assess the association between HARP and progression-free survival (PFS), defined as the time from initiation of CCRT to local, regional, or distant disease progression, death, or last follow-up. Continuous variables were summarized as medians with ranges, and categorical variables as frequencies and percentages. Continuous variables were assessed for normality before analysis and compared using Student’s *t*-test or appropriate non-parametric tests, as indicated, whereas categorical variables were compared using the chi-square test. Spearman correlation analysis was applied where relevant.

The prognostic association between HARP and survival outcomes was first evaluated as a continuous variable using Cox proportional hazards regression models. Restricted cubic spline (RCS) Cox regression analyses were additionally performed to explore potential nonlinear associations between HARP values and survival outcomes. Survival outcomes were estimated using the Kaplan–Meier method and compared using the log-rank test. For exploratory clinical risk stratification, receiver operating characteristic (ROC) curve analyses using the Youden index were performed to identify candidate HARP cut-off values for PFS and OS. Given the close proximity of the outcome-specific thresholds, a unified exploratory HARP cut-off value was selected for subsequent categorical survival analyses. Because this threshold was derived and evaluated within the same study cohort, the categorical analyses were considered exploratory and hypothesis-generating rather than internally validated.

Variables demonstrating statistical significance or a trend toward significance (*p* < 0.10) in univariate analyses were entered into multivariable Cox proportional hazards regression models to identify independent prognostic factors. The proportional hazards assumption was assessed using Schoenfeld residual-based methods and was not violated for the final multivariable Cox models. Because HARP is mathematically derived from hemoglobin, Alb, and CRP, comparative analyses versus hemoglobin and the Alb-to-CRP (AC) ratio were performed using separate multivariable Cox models adjusted for identical clinical covariates to avoid collinearity arising from shared components; these results are presented in Supplementary Table S1. Internal validation of the multivariable Cox models was performed using bootstrap resampling with 1000 repetitions. Model discrimination was assessed using Harrell’s concordance index (C-index), and optimism-corrected estimates were derived to evaluate model stability and potential overfitting. Given the censored time-to-event nature of OS and PFS, formal pairwise comparisons of model discrimination were performed between the HARP model and the corresponding Hb and AC models using correlated Harrell C-index estimates derived from the same patients. Differences in apparent C-index (ΔC) were calculated as C-index*_HARP_* − C-index*_comparator_*, with positive values favoring HARP. Ninety-five percent confidence intervals and two-sided *p* values for ΔC were obtained while accounting for the correlation between model-specific C-index estimates. All statistical analyses were performed using IBM SPSS Statistics version 26.0 (IBM Corp., Armonk, NY, USA) and R software version 4.5.1 (R Foundation for Statistical Computing, Vienna, Austria). All hypothesis-driven statistical tests were two-tailed, and statistical significance was defined as *p* < 0.05.

## 3. Results

This retrospective cohort analysis included 248 patients who met the eligibility criteria (Table 1). The median age was 59 years (range, 23–79 years), and 197 patients (79.4%) were male. The majority of patients had advanced disease, with T3–4 disease observed in 202 patients (81.5%) and N2–3 disease in 194 patients (78.2%). Overall, 177 patients (71.4%) completed three cycles of concurrent chemotherapy, and 178 (71.8%) received the recommended two cycles of adjuvant chemotherapy. No treatment-related deaths occurred during concurrent chemoradiotherapy. However, three patients (1.2%) experienced late treatment-related mortality: one due to aspiration pneumonia secondary to tracheoesophageal fistula at 8 months, one due to intractable carotid artery blowout at 19 months, and one due to refractory temporal lobe necrosis at 27 months of follow-up.

At a median follow-up of 85.7 months (95% CI, 67.9–103.5), estimated using the reverse Kaplan–Meier method, the median PFS and OS for the entire cohort were 66.0 months (95% CI, 53.4–78.6) and 108.0 months (95% CI, 92.1–123.8), respectively. The fact that the median OS exceeded the reverse Kaplan–Meier estimate of median follow-up does not represent an inconsistency, because these quantities characterize the survival and censoring distributions, respectively, and are not constrained to occur in the same numerical order. The 5-year and 10-year PFS rates were 54.0% and 33.0%, whereas the corresponding OS rates were 76.9% and 44.4% (Table 2).

**Figure 1:**
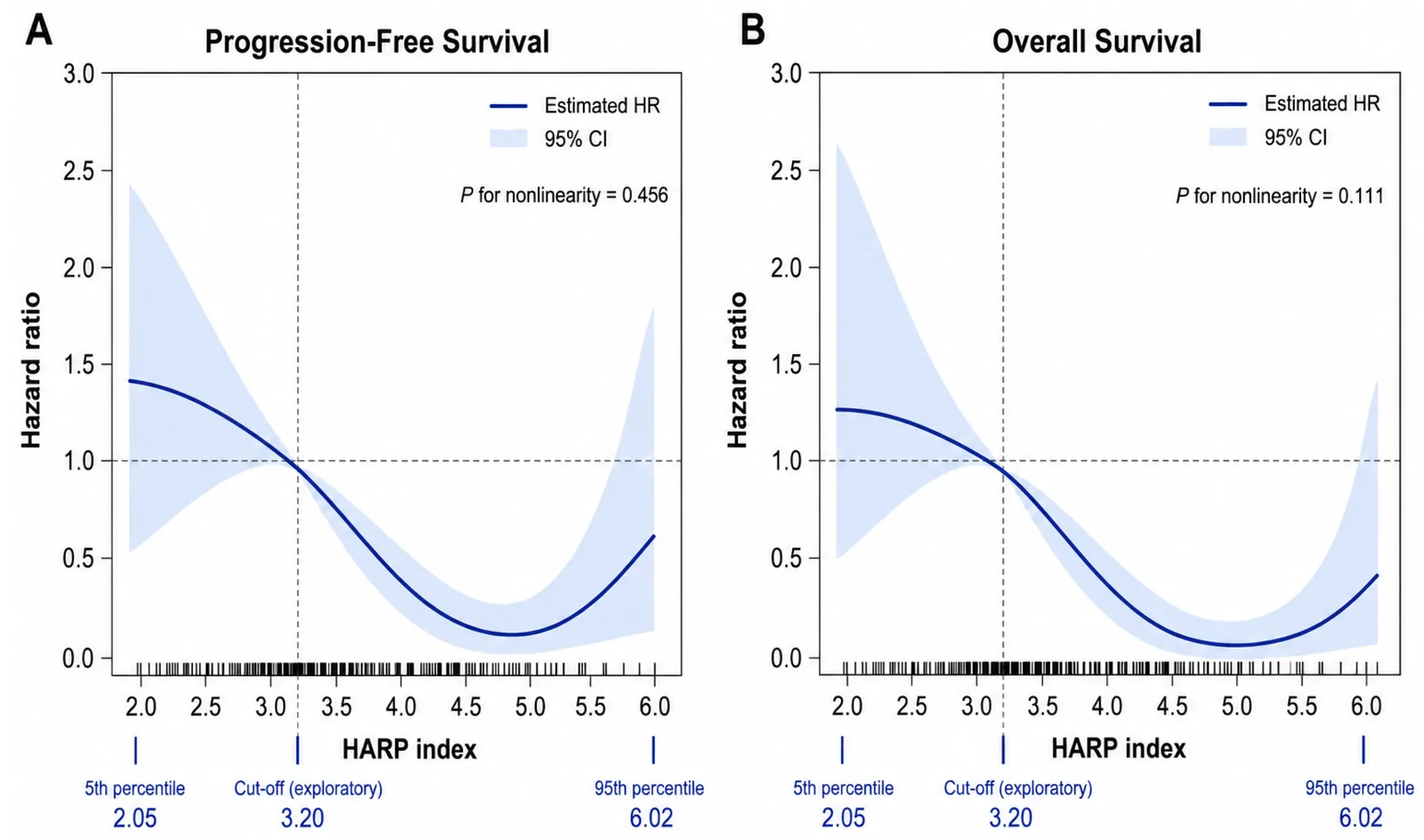
Restricted cubic spline analyses of continuous HARP values for progression-free and overall survival. Note: (**A**) Association between continuous HARP values and hazard ratio for progression or death (PFS endpoint). (**B**) Association between continuous HARP values and hazard ratio for death (OS endpoint). Solid lines represent estimated hazard ratios, and shaded areas indicate 95% confidence intervals. Vertical dashed lines denote the exploratory HARP cut-off value of 3.2, whereas horizontal dashed lines indicate HR = 1. Rug marks along the *x*-axis represent the distribution of observed HARP values. Curves are displayed across the 5th–95th percentile range of HARP to reduce sparse-tail overinterpretation. Abbreviations: CI, confidence interval; HARP, hemoglobin–albumin–C-reactive protein; HR, hazard ratio; OS, overall survival; PFS, progression-free survival.

When analyzed as a continuous variable, higher HARP values were significantly associated with improved survival outcomes. In continuous Cox regression analyses, each one-unit increase in HARP was associated with lower risks of mortality (HR, 0.464; 95% CI, 0.355–0.606; *p* < 0.001) and disease progression (HR, 0.712; 95% CI, 0.603–0.840; *p* < 0.001). Restricted cubic spline analyses demonstrated a consistent inverse association between HARP and survival outcomes without statistically significant evidence of nonlinearity for either OS (P for nonlinearity = 0.111) or PFS (P for nonlinearity = 0.456) (Figure 1). Detailed results of the continuous Cox and spline analyses are summarized in Supplementary Table S2.

Bootstrap internal validation using 1000 resamples demonstrated stable model discrimination, with optimism-corrected C-index values of 0.680 for OS and 0.594 for PFS. Bootstrap-derived hazard ratio estimates for continuous HARP remained directionally consistent for both endpoints, further supporting the internal stability of the observed prognostic associations (Supplementary Table S3).

Formal pairwise comparisons of model discrimination further demonstrated that the HARP model had numerically higher apparent C-indices than both comparator models for each endpoint (Supplementary Table S4). For OS, the apparent C-index was 0.704 for HARP, compared with 0.676 for Hb (ΔC, 0.028; 95% CI, −0.003 to 0.059; *p* = 0.078) and 0.655 for AC (ΔC, 0.049; 95% CI, 0.018–0.080; *p* = 0.002). Thus, HARP demonstrated significantly greater discrimination than AC but not Hb. For PFS, the corresponding apparent C-indices were 0.614, 0.594, and 0.575, respectively. The difference between HARP and Hb was not statistically significant (ΔC, 0.020; 95% CI, −0.005 to 0.045; *p* = 0.118), whereas HARP demonstrated significantly greater discrimination than AC (ΔC, 0.039; 95% CI, 0.009–0.069; *p* = 0.011).

**Figure 2:**
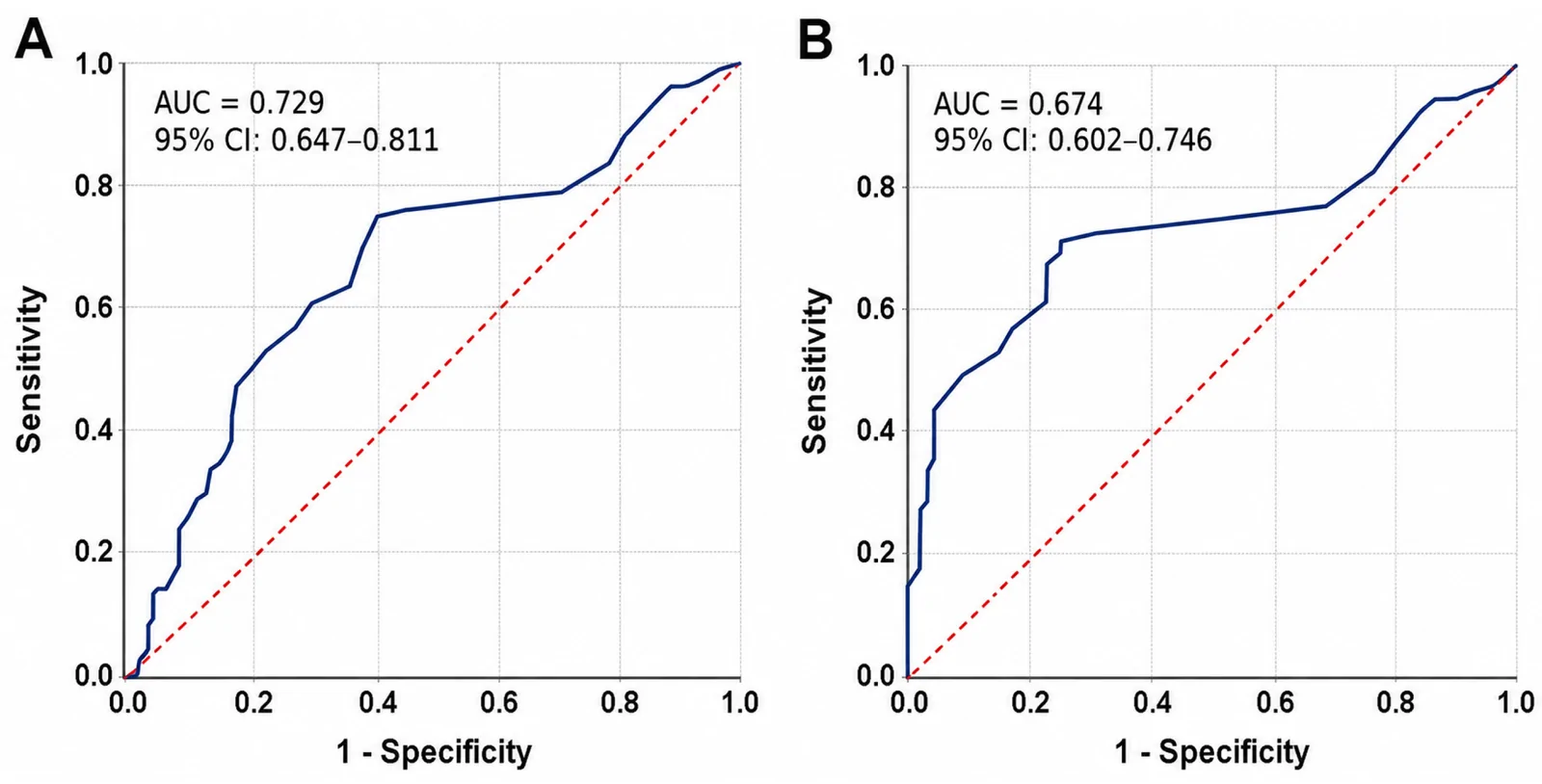
Receiver operating characteristic curve analysis according to pre-treatment HARP index values. Note: Receiver operating characteristic (ROC) curve analyses evaluating the prognostic performance of the pre-treatment hemoglobin–albumin–C-reactive protein (HARP) index for predicting (**A**) overall survival (OS) and (**B**) progression-free survival (PFS) in patients with locally advanced nasopharyngeal carcinoma. The exploratory HARP cut-off value (3.2) was identified using ROC analysis and subsequently applied for risk stratification in survival analyses. The area under the curve (AUC) was 0.729 for OS and 0.674 for PFS, indicating acceptable discriminative performance. ROC analyses were performed using standard nonparametric methods.

For exploratory clinical stratification, ROC curve analyses were performed to identify candidate HARP cut-off values. Using the Youden index, the identified cut-off values were 3.17 for PFS (AUC, 0.674; sensitivity, 71.6%; specificity, 67.6%; J-index, 0.392) and 3.24 for OS (AUC, 0.729; sensitivity, 72.6%; specificity, 70.3%; J-index, 0.429). Because these thresholds were closely aligned, a unified exploratory cut-off value of 3.2 was selected for subsequent categorical survival analyses to facilitate clinical interpretation (Figure 2). Because this threshold was derived within the same study cohort, it was intended solely for exploratory risk stratification rather than as an internally validated clinical cut-off. Separately, five-year time-dependent ROC analyses demonstrated moderate discrimination for OS and modest discrimination for PFS, with AUC values of 0.674 and 0.601, respectively.

**Figure 3:**
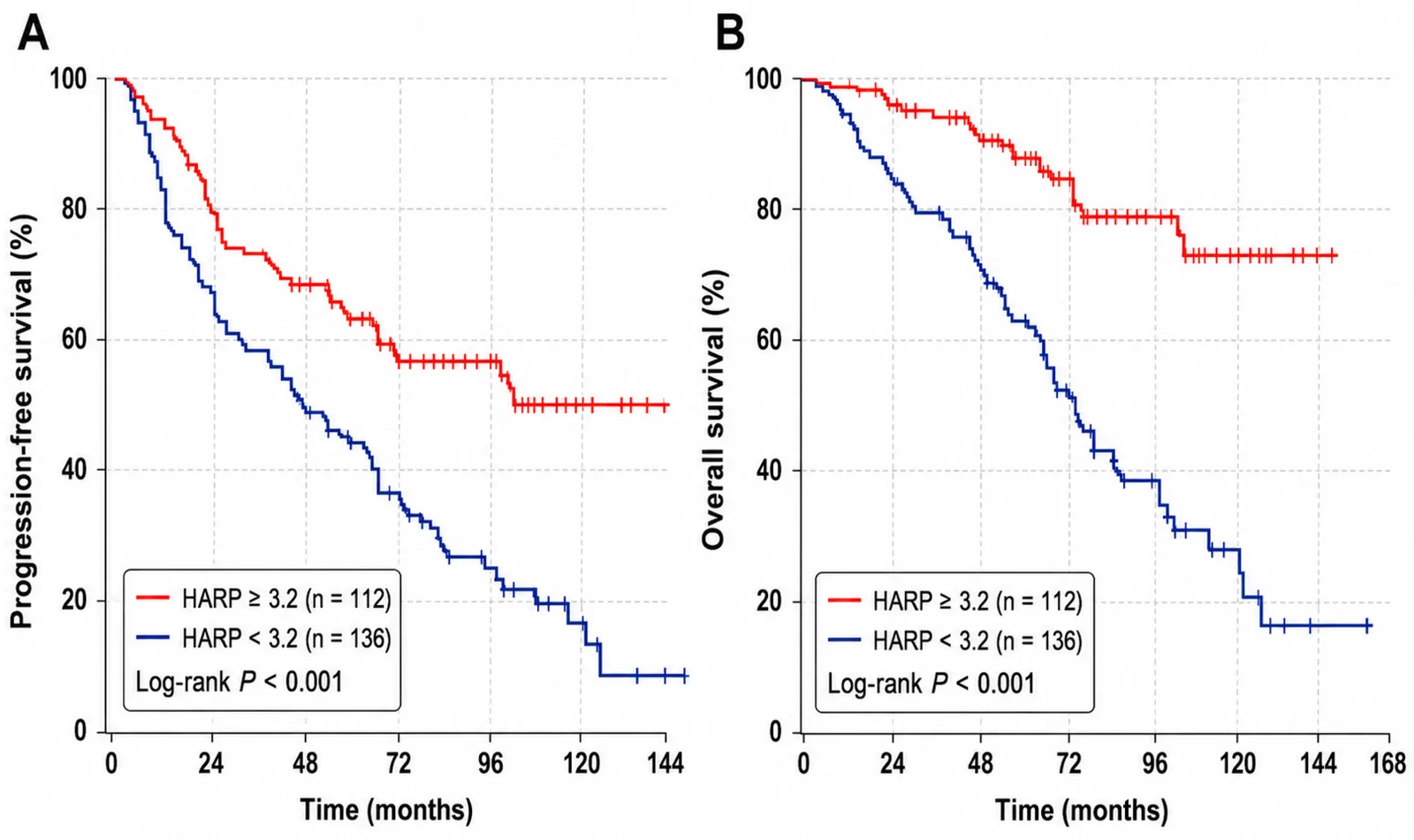
Kaplan–Meier survival curves according to pretreatment HARP index. (**A**) Progression-free survival stratified by HARP ≥ 3.2 versus HARP < 3.2. (**B**) Overall survival stratified by HARP ≥ 3.2 versus HARP < 3.2. Survival distributions were compared using the log-rank test. Tick marks indicate censored observations.

Baseline characteristics were generally balanced between patients with HARP < 3.2 (n = 136) and HARP ≥ 3.2 (n = 112), with no statistically significant differences in age, sex, ECOG performance status, histological subtype, T-stage, N-stage, concurrent chemotherapy completion, or adjuvant chemotherapy use (all *p* > 0.05; Table 1). Patients with HARP ≥ 3.2 experienced significantly better survival outcomes than those with HARP < 3.2. Median PFS was not reached in the HARP ≥ 3.2 cohort, compared with 47.0 months (95% CI, 32.7–61.3) in the HARP < 3.2 cohort (*p* = 0.001). The corresponding 5-year and 10-year PFS rates were 62.9% and 50.7% for patients with HARP ≥ 3.2, compared with 43.3% and 16.7% for those with HARP < 3.2. Similarly, median OS was not reached in the HARP ≥ 3.2 cohort, compared with 72.0 months (95% CI, 62.1–81.9) in the HARP < 3.2 cohort (*p* < 0.001). The corresponding 5-year and 10-year OS rates were 89.3% and 74.2%, respectively, compared with 62.3% and 19.9%, respectively (Table 2 and Table 3, Figure 3).

## 4. Discussion

This retrospective analysis evaluated the prognostic relevance of the novel HARP index in patients with LANPC receiving definitive CCRT. To our knowledge, this investigation is the first to evaluate a biologically designed composite index integrating Hb, Alb, and CRP as a continuous prognostic biomarker in patients with LANPC receiving definitive CCRT. Our findings demonstrated that lower pretreatment HARP values were independently associated with inferior PFS and OS, both when analyzed as a continuous variable and when dichotomized at an empirically derived exploratory cut-off value of 3.2. Importantly, these associations remained statistically significant after adjustment for established prognostic factors, including T-stage and N-stage.

Recent years have seen a growing emphasis on composite biomarkers that integrate systemic inflammation, nutritional status, and host-related parameters to refine prognostic stratification in NPC. Emerging evidence indicates that indices such as the prognostic nutritional index (PNI), systemic immune-inflammation index (SII), and platelet-to-Ab ratio confer independent prognostic value beyond conventional TNM staging in patients managed with modern chemoradiotherapy. For example, Jiang et al. demonstrated that PNI independently predicted both OS and PFS, thereby improving risk discrimination among patients with NPC treated with IMRT [27]. In parallel, Xiong et al. showed that integrating SII with Epstein–Barr virus DNA status improved prognostic stratification in locoregionally advanced disease [28]. More recently, Hua et al. affirmed that inflammation–nutrition-based ratios incorporating Alb were independently associated with survival outcomes in patients receiving CCRT [29]. Collectively, these contemporary findings provide a compelling framework for developing integrative host-related prognostic indices, such as HARP, in LANPC.

Consistent with the previous literature, our findings reaffirm the prognostic significance of T-stage and N-stage in LANPC, both of which remain foundational components of the universally adopted TNM staging system [9]. Nevertheless, the TNM framework is principally anchored in tumor-centric metrics—namely local invasion and nodal dissemination—while their extent and laterality are systematically codified [11,12,13]. Importantly, accumulating evidence shows that patients sharing identical TN categories may nonetheless experience profoundly divergent clinical trajectories, with some exhibiting survival patterns resembling those of metastatic disease and others achieving outcomes comparable to those of earlier-stage counterparts [14,15]. For example, Balsak et al. reported a median OS of 181 months (95% CI, 57.6–304.4 months) in a cohort of patients with NPC, underscoring the substantial heterogeneity in long-term outcomes even among comparably staged populations [30]. This variability likely arises, at least in part, from the inherent limitations of anatomical staging in capturing important host-related biological determinants—including tumor oxygenation, nutritional status, systemic inflammation, and immune competence—that critically influence therapeutic response and prognosis. These considerations formed the conceptual basis for the present investigation, in which we hypothesized that integrating host-related parameters alongside TNM staging could yield a more nuanced and clinically actionable framework for prognostic stratification in LANPC.

The principal contribution of the present investigation to the LANPC literature is the identification of the novel HARP index as a robust and independently validated prognostic biomarker. Lower pretreatment HARP values, evaluated as both continuous and categorical variables, were consistently associated with inferior survival outcomes. Specifically, patients with HARP < 3.2 had substantially shorter median PFS (47.0 months vs. not reached; *p* < 0.001) and OS (72.0 months vs. not reached; *p* < 0.001) than those with HARP ≥ 3.2. Furthermore, the markedly lower 10-year PFS (16.7% vs. 50.7%) and OS rates (19.9% vs. 74.2%) observed in the low-HARP cohort underscore the persistent adverse prognostic impact associated with reduced HARP values. Importantly, continuous Cox proportional hazards and restricted cubic spline analyses demonstrated a stable inverse association between HARP and adverse clinical outcomes, while bootstrap-based internal validation confirmed preserved model discrimination with minimal optimism, supporting the stability and internal consistency of the observed prognostic associations. The categorical analyses should be interpreted in the context of their exploratory nature. Because the HARP threshold was derived using the Youden index within the same cohort in which survival was subsequently evaluated, the observed hazard ratios may be optimistically estimated. Accordingly, the continuous analyses constitute the primary evidence supporting the prognostic association of HARP, whereas the categorical analyses are intended to facilitate clinical interpretation and generate hypotheses for future validation. Independent external validation will be required before a fixed clinical threshold can be recommended.

Although direct cross-study comparisons are limited by the absence of prior evaluations of HARP in any malignancy, the present findings are biologically and clinically consistent with the established prognostic significance of its constituent parameters. Comparative analyses provided a more nuanced assessment of the discriminatory performance of HARP relative to its component-based comparators. In parallel multivariable models adjusted for identical clinical covariates, HARP demonstrated significantly greater discrimination than the AC ratio for both OS and PFS (Supplementary Table S4). Although its C-index was also numerically higher than that of Hb for both endpoints, these differences did not reach statistical significance. Accordingly, the present findings support greater discrimination of HARP relative to AC but do not establish statistical superiority over Hb. These results suggest that incorporating Hb into an inflammation–nutrition-based construct may enhance prognostic discrimination relative to AC alone; however, whether the composite formulation provides meaningful incremental value beyond Hb itself remains unresolved and requires evaluation in independent cohorts. Biologically, HARP integrates complementary domains of oxygen-carrying capacity, nutritional status, and systemic inflammation. Building on recent advances in multidimensional biomarker research [27,28,29], HARP extends existing inflammation- and nutrition-based indices by incorporating Hb as a surrogate marker of tumor hypoxia and radiosensitivity [21,22,23,31,32], thereby integrating radiobiological information into a host-related prognostic framework. This conceptual approach aligns with contemporary efforts to refine prognostic stratification in NPC using composite rather than isolated laboratory biomarkers [27,28,29].

Consistent with this interpretation, Topkan et al. demonstrated that pretreatment Hb levels below 11.0 g/dL were independently associated with diminished locoregional control, PFS, and OS in patients with LANPC [14], findings subsequently corroborated by Cobanoglu et al. [33] and by Guo et al., who confirmed the continued prognostic relevance of Hb in the IMRT era [34]. Similarly, investigations evaluating inflammation- and nutrition-based biomarkers have consistently shown that elevated pretreatment CAR values are associated with significantly inferior survival outcomes in NPC, including reduced 5-year OS [17] and poorer OS as well as distant metastasis-free survival in meta-analytic evidence [18]. Given that HARP integrates Hb with the inverse of CAR—that is, the Alb-to-CRP (AC) ratio—these convergent findings provide a strong biological rationale for the prognostic performance of HARP observed in the present study. Nevertheless, incorporation of HARP alongside TNM staging into routine clinical practice requires confirmation through prospective multicenter investigations and external validation studies.

The precise biological mechanisms underlying the association between the novel HARP index and prognosis in LANPC remain to be fully elucidated. Nevertheless, the constituent components of HARP reflect distinct yet complementary dimensions of tumor biology and host response. Hemoglobin reflects systemic oxygen-carrying capacity and serves as an indirect surrogate of tumor hypoxia, a well-established mediator of radioresistance and adverse clinical outcomes in NPC and other head and neck malignancies [31,32]. In contrast, serum Alb and CRP are widely recognized markers of nutritional reserve, systemic inflammation, and immune competence, all of which substantially influence treatment tolerance, tumor aggressiveness, and long-term survival [35,36,37,38]. By integrating these parameters into a single composite biomarker, HARP may provide a more comprehensive representation of the complex interplay between tumor biology and host physiology than any individual component alone. Thus, although HARP itself represents a novel prognostic construct, its biological plausibility is strongly supported by the established prognostic significance of its constituent parameters. This concordance with existing evidence not only reinforces the credibility of the present findings but also positions HARP as a promising candidate for further validation in large-scale multicenter prospective studies.

The novelty of the present study should not be interpreted as arising solely from the mathematical combination of existing laboratory variables. Rather, HARP was developed as a biologically motivated composite index based on predefined principles that integrate oxygen-carrying capacity, nutritional reserve, and systemic inflammation into a single interpretable framework. Moreover, unlike our previous biomarker investigations, which relied more heavily on categorical risk stratification, the present study evaluated HARP principally as a continuous biomarker using multivariable Cox regression and restricted cubic spline analyses, allowing characterization of prognostic risk across the full biological spectrum. Accordingly, the principal methodological contribution of this study lies in both the biological construction of the index and its continuous analytical framework.

An important consideration regarding the novel HARP index is the extent to which it fulfills the characteristics desirable for a clinically useful prognostic biomarker. In general, an effective prognostic indicator should reliably predict disease outcomes while remaining economically feasible, readily accessible through routine clinical testing, straightforward to calculate, reproducible across different institutional settings, and broadly applicable to heterogeneous patient populations [39]. Within this framework, the HARP index exhibits several favorable attributes: it is based on universally available laboratory parameters, is easy to calculate, is low-cost, and demonstrated independent prognostic significance across multiple analytic approaches. However, any consideration of its clinical utility must be tempered by the absence of pretreatment EBV DNA data in the present analysis. Although HARP remained independently associated with survival outcomes after adjustment for the available clinicopathologic factors, its incremental prognostic value beyond EBV DNA remains unestablished. Rigorous prospective multicenter validation evaluating HARP within models incorporating EBV DNA and established clinicopathologic factors is therefore essential before its role in clinical risk stratification can be defined. Until such evidence is available, endorsement of HARP for routine clinical use would be premature and is not supported by the present study.

Despite rigorous inclusion criteria and a largely standardized treatment protocol, several methodological limitations of the present investigation warrant consideration. First, the retrospective single-center design introduces unavoidable risks of selection bias and residual confounding, potentially limiting the external validity and generalizability of the findings. Because the study cohort was derived from an institutional database that has also supported previous retrospective biomarker investigations, partial overlap with earlier studies is unavoidable. However, the present study addressed a distinct biological hypothesis using a different composite biomarker and an independent analytical framework centered on continuous risk modeling. Nevertheless, repeated biomarker analyses within partially overlapping cohorts may increase the risk of selective findings, and the present results should therefore be interpreted cautiously. Consequently, confirmation in independent external cohorts remains essential before broad clinical application. Second, HARP was calculated from a single pretreatment blood sample, precluding assessment of longitudinal changes in oxygenation status, nutritional reserve, and systemic inflammatory burden during treatment and follow-up. Accordingly, future prospective studies incorporating serial HARP measurements may provide additional prognostic insights. Third, although patients with active infection, immunosuppressive disease, and recent immunosuppressive medication use were excluded to minimize non-tumor-related inflammatory variability, residual confounding from unmeasured inflammatory, nutritional, hepatic, renal, or comorbidity-related factors cannot be fully excluded in this retrospective design. Fourth, because HARP is mathematically derived from hemoglobin, Alb, and CRP, simultaneous inclusion of these correlated variables within a single multivariable model would introduce collinearity and potentially distort effect estimates; therefore, comparative analyses using separate models were considered the most statistically appropriate approach. Fifth, although bootstrap-based internal validation demonstrated stable model performance with minimal optimism, rigorous external validation in independent multicenter cohorts remains necessary before broader clinical implementation of HARP can be considered. Sixth, pretreatment plasma Epstein–Barr virus (EBV) DNA, one of the most important prognostic biomarkers in NPC, was not routinely available throughout the study period and therefore could not be incorporated into the present analyses. Consequently, we were unable to determine whether HARP provides incremental prognostic information beyond EBV DNA or whether combining these biomarkers would further improve risk stratification. Future prospective studies should evaluate the complementary prognostic value of HARP alongside EBV DNA and other established biomarkers. Finally, although HARP demonstrated robust prognostic associations, the observational design of the present study precludes conclusions regarding the clinical utility of HARP-guided therapeutic decision-making. Whether incorporation of HARP into treatment selection improves patient outcomes should be evaluated in prospective interventional studies.

## 5. Conclusions

In conclusion, this investigation identifies the HARP index as a biologically plausible and independently associated prognostic biomarker in patients with LANPC receiving definitive CCRT. Lower pretreatment HARP values were consistently associated with adverse survival outcomes, highlighting its potential as a low-cost and accessible adjunct to conventional clinicopathologic risk assessment. Nonetheless, its incremental prognostic value beyond EBV DNA remains undetermined. Rigorous prospective multicenter studies incorporating EBV DNA, with formal assessment of the incremental prognostic value of HARP, together with external validation, are required before a fixed HARP threshold can be recommended or the index can be integrated into routine clinical practice. Until such evidence is available, HARP should be regarded as an investigational prognostic tool rather than an established component of clinical risk stratification.

## Figures and Tables

**Table 1:** Pretreatment and treatment characteristics.

Characteristics	Whole Cohort (N = 248)	HARP < 3.2 (N = 136)	HARP ≥ 3.2 (N = 112)	*p*-Value
Median age, years (range)	59 (23–79)	59 (23–79)	58 (27–79)	0.86
Age group (N; %)				0.78
≥65 years	82 (33.1)	46 (33.8)	36 (32.1)	
<65 years	166 (66.9)	90 (66.2)	76 (67.9)	
Gender (N; %)				0.32
Male	197 (79.4)	105 (77.2)	92 (82.1)	
Female	51 (20.6)	31 (22.8)	20 (17.9)	
ECOG performance (N; %)				0.54
0	118 (47.6)	66 (48.5)	52 (46.4)	
1	130 (52.4)	70 (51.5)	60 (53.6)	
WHO histology (N; %)				0.69
2	33 (13.3)	18 (13.2)	15 (13.4)	
3	215 (86.7)	118 (86.8)	97 (86.6)	
T-stage (N; %)				0.72
1–2	46 (18.5)	26 (19.1)	20 (17.9)	
3–4	202 (81.5)	110 (80.9)	92 (82.1)	
N-stage (N; %)				0.83
0–1	54 (21.8)	30 (22.1)	24 (21.4)	
2–3	194 (78.2)	106 (77.9)	88 (78.6)	
Concurrent chemotherapy cycles (N; %)				0.92
1–2	71 (28.6)	39 (28.7)	32 (28.6)	
3	177 (71.4)	97 (71.3)	80 (71.4)	
Adjuvant chemotherapy cycles (N; %)				0.37
0	70 (28.2)	40 (29.4)	30 (26.8)	
1–2	178 (71.8)	96 (70.6)	82 (73.2)	

Abbreviations: HARP, hemoglobin–albumin–C-reactive protein; ECOG: Eastern Cooperative Oncology Group; WHO: World Health Organization; T-stage: Tumor stage; N-stage: Nodal stage.

**Table 2:** Survival results for the whole study cohort and per HARP group.

Outcome	Whole Cohort (N = 248)	HARP < 3.2 (N = 136)	HARP ≥ 3.2 (N = 112)	*p*-Value
Progression-free survival				0.001
Median; mo. (95% CI)	66.0 (53.4–78.6)	47.0 (32.7–61.3)	NR	
5-year rate (%)	54.0	43.3	62.9	
10-year rate (%)	33.0	16.7	50.7	
Overall survival				<0.001
Median; mo. (95% CI)	108.0 (92.1–123.8)	72.0 (62.1–81.9)	NR	
5-year rate (%)	76.9	62.3	89.3	
10-year rate (%)	44.4	19.9	74.2	

Abbreviations: HARP, hemoglobin–albumin–C-reactive protein; CI, confidence interval; mo, months; NR, not reached.

**Table 3:** Results of univariate and multivariable Cox proportional hazards regression analyses for progression-free survival and overall survival.

Factor	PFS Univariate *p*-Value	PFS Univariate HR (95% CI)	PFS Multivariate *p*-Value	PFS Multivariate HR (95% CI)	OS Univariate *p*-Value	OS Univariate HR (95% CI)	OS Multivariate *p*-Value	OS Multivariate HR (95% CI)
Age group (<65 vs. ≥65 y)	0.64	1.03 (0.62–1.45)	–	–	0.72	1.08 (0.71–1.37)	–	–
Gender (Male vs. Female)	0.77	0.88 (0.52–1.24)	–	–	0.67	0.91 (0.57–1.24)	–	–
ECOG (0 vs. 1)	0.82	1.07 (0.61–1.54)	–	–	0.79	1.16 (0.83–1.49)	–	–
WHO histology (2 vs. 3)	0.87	0.97 (0.86–1.08)	–	–	0.89	0.92 (0.79–1.05)	–	–
T-stage (3–4 vs. 1–2)	0.010	1.52 (1.12–2.33)	0.024	1.45 (1.09–2.22)	0.005	1.61 (1.23–2.33)	0.016	1.72 (1.15–2.86)
N-stage (2–3 vs. 0–1)	<0.001	1.56 (1.30–2.44)	0.001	1.72 (1.28–2.70)	0.001	1.85 (1.43–2.56)	0.001	2.27 (1.45–4.55)
Concurrent chemotherapy cycles (3 vs. 1–2)	0.32	0.82 (0.52–1.12)	–	–	0.26	0.86 (0.67–1.06)	–	–
Adjuvant chemotherapy cycles (1–2 vs. 0)	0.36	0.73 (0.44–1.02)	–	–	0.33	0.79 (0.43–1.15)	–	–
HARP (<3.2 vs. ≥3.2)	<0.001	4.42 (2.04–6.81)	<0.001	3.86 (2.27–6.49)	<0.001	3.92 (1.78–6.06)	<0.001	3.06 (1.89–5.12)

Abbreviations: PFS, progression-free survival; OS, overall survival; HR, hazard ratio; CI, confidence interval; ECOG, Eastern Cooperative Oncology Group; WHO, World Health Organization; HARP, hemoglobin–albumin–C-reactive protein index.

## Data Availability

The patient-level data generated and analyzed in this study are not publicly available because the institutional ethics approval governing the study does not permit deposition of anonymized patient-level data in public repositories. Any request for access to the underlying data is subject to applicable institutional and ethics committee requirements and cannot be granted solely at the discretion of the authors.

## Supplementary Materials

The following supporting information can be downloaded at: https://www.mdpi.com/article/10.3390/diseases14090306/s1, Supplementary Table S1: Comparative multivariable Cox regression models evaluating the prognostic performance of HARP and its constituent components; Supplementary Table S2: Continuous Cox proportional hazards and restricted cubic spline analyses of HARP for progression-free and overall survival; Supplementary Table S3: Bootstrap internal validation of continuous Cox regression models for overall and progression-free survival; Supplementary Table S4: Comparative multivariable Cox regression models incorporating HARP, hemoglobin, or the albumin-to-C-reactive protein ratio.

## Appendix A

**Table A1:** STROBE Checklist for Observational Cohort Studies.

Item No.	STROBE Recommendation	Reported in Manuscript (Section/Page)
Title and Abstract		
1a	Indicate the study’s design with a commonly used term in the title or abstract	Title; Abstract
1b	Provide an informative and balanced summary of what was done and what was found	Abstract
Introduction		
2	Explain the scientific background and rationale for the investigation	Introduction
3	State specific objectives, including any prespecified hypotheses	Introduction
Methods		
4	Present key elements of study design early in the paper	Study Design and Participants
5	Describe the setting, locations, and relevant dates	Study Design and Participants
6a	Give eligibility criteria and sources/methods of selection of participants	Study Design and Participants
6b	For matched studies, give matching criteria and number of exposed/unexposed	Not applicable (no matching)
7	Clearly define all outcomes, exposures, predictors, confounders, and effect modifiers	Measurement of the HARP Index; Clinical Endpoints and Statistics
8	Give sources of data and details of methods of assessment	Measurement of the HARP Index
9	Describe any efforts to address potential sources of bias	Study Design and Participants; Limitations
10	Explain how the study size was arrived at	Study Design and Participants
11	Explain how quantitative variables were handled in the analyses	Clinical Endpoints and Statistics
12a	Describe all statistical methods, including those used to control for confounding	Clinical Endpoints and Statistics
12b	Describe any methods used to examine subgroups and interactions	Clinical Endpoints and Statistics
12c	Explain how missing data were addressed	Clinical Endpoints and Statistics
12d	If applicable, explain how loss to follow-up was addressed	Not applicable
12e	Describe any sensitivity analyses	Not applicable
Results		
13a	Report numbers of individuals at each stage of study	Results
13b	Give reasons for non-participation at each stage	Results
13c	Consider use of a flow diagram	Results
14a	Give characteristics of study participants	Table 1
14b	Indicate number of participants with missing data	Results
15	Report numbers of outcome events or summary measures	Results
16a	Give unadjusted and adjusted estimates with precision	Table 3
16b	Report category boundaries when continuous variables were categorized	Clinical Endpoints and Statistics; Results
16c	Translate estimates of relative risk into absolute risk	Results
Discussion		
17	Summarize key results with reference to study objectives	Discussion
18	Discuss limitations of the study	Limitations
19	Give a cautious overall interpretation of results	Discussion
20	Discuss generalizability of the study results	Discussion
Other Information		
21	Give the source of funding and role of funders	Funding Statement
