# Supplementary material for: The HARP (Hemoglobin–Albumin–C-Reactive Protein) Index: A Biologically Designed Composite Biomarker for Continuous Prognostic Modeling in Locally Advanced Nasopharyngeal Carcinoma
Source: Diseases. 2026 Aug 25;14(9):306. doi: 10.3390/diseases14090306 (PMC13605612; doi:10.3390/diseases14090306)
Supplement: Supplementary file 1 [file diseases-14-00306-s001.zip › diseases-4474502-supplementary.pdf]

Supplementary Tables

Supplementary Table S1. Comparative multivariable Cox regression models evaluating the prognostic performance of HARP and its constituent components

Panel A. Progression-free survival (PFS)

Model	Biomarker	Adjusted covariates	Adjusted HR (95% CI)	P-value	C-index
A	HARP (<3.2 vs ≥3.2)	T-stage; N-stage	3.86 (2.27–6.49)	<0.001	0.689
B	Hemoglobin (Hb) (<11 vs ≥11 g/dL)	T-stage; N-stage	2.01 (1.56–3.87)	<0.001	0.608
C	AC ratio (Albumin/CRP) (per 1 SD decrease)	T-stage; N-stage	2.28 (1.64–3.75)	0.001	0.567

Panel B. Overall survival (OS)

Model	Biomarker	Adjusted covariates	Adjusted HR (95% CI)	P-value	C-index
A	HARP (<3.2 vs ≥3.2)	T-stage; N-stage	3.06 (1.89–5.12)	<0.001	0.677
B	Hemoglobin (Hb) (<11 vs ≥11 g/dL)	T-stage; N-stage	2.17 (1.73–2.78)	<0.001	0.616
C	AC ratio (Albumin/CRP) (per 1 SD decrease)	T-stage; N-stage	1.98 (1.51–2.97)	0.003	0.562

Notes: Each biomarker was evaluated in a separate multivariable Cox proportional hazards model to avoid collinearity arising from shared components. Previously reported clinically relevant thresholds were used for hemoglobin analyses. Hazard ratios for the AC ratio are reported per 1-standard deviation decrease to facilitate interpretability and comparability across models. Model discrimination was assessed using Harrell's concordance index (C-index).

Abbreviations: AC, albumin-to-C-reactive protein; CI, confidence interval; CRP, C-reactive protein; HARP, hemoglobin–albumin–C-reactive protein; Hb, hemoglobin; HR, hazard ratio; OS, overall survival; PFS, progression-free survival

Supplementary Table S2. Continuous Cox proportional hazards and restricted cubic spline analyses of HARP for progression-free and overall survival.

Outcome	HR per 1-unit HARP increase	95% CI	P-value	Nonlinearity P
OS	0.464	0.355–0.606	<0.001	0.111
PFS	0.712	0.603–0.840	<0.001	0.456

Note: Hazard ratios represent the relative risk associated with each 1-unit increase in the HARP index. Nonlinearity was assessed using restricted cubic spline Cox regression models.

Abbreviations: CI, confidence interval; HARP, hemoglobin–albumin–C-reactive protein; HR, hazard ratio; OS, overall survival; PFS, progression-free survival

Supplementary Table S3. Bootstrap internal validation of continuous Cox regression models for overall and progression-free survival

Outcome	n	Events	Apparent C-index	Optimism	Optimism-corrected C-index	Bootstrap HR median	Bootstrap 95% CI
OS	248	88	0.680	< 0.001	0.680	0.461	0.352–0.571
PFS	248	144	0.595	0.001	0.594	0.707	0.590–0.830

Note: Internal validation was performed using 1000 bootstrap resamples. Optimism-corrected C-index values represent bootstrap-adjusted model discrimination estimates. Bootstrap hazard ratios correspond to the median resampled HR estimates for continuous HARP.

Abbreviations: CI, confidence interval; HARP, hemoglobin–albumin–C-reactive protein; HR, hazard ratio; OS, overall survival; PFS, progression-free survival.

Supplementary Table S4. Comparative multivariable Cox regression models incorporating HARP, hemoglobin, or the albumin-to-C-reactive protein ratio.

Model	Apparent C-index	Optimism-corrected C-index	ΔC (HARP − comparator)	95% CI for ΔC	P value
Overall survival					
HARP model	0.704	0.680	Reference	—	—
Hb model	0.676	0.657	**0.028**	−0.003 to 0.059	0.078
AC model	0.655	0.638	**0.049**	0.018 to 0.080	0.002
Progression-free survival					
HARP model	0.614	0.594	Reference	—	—
Hb model	0.594	0.582	**0.020**	−0.005 to 0.045	0.118
AC model	0.575	0.563	**0.039**	0.009 to 0.069	0.011

Note: Apparent C-indices were calculated from the fitted multivariable Cox models. Formal pairwise comparisons of apparent model discrimination accounted for the correlation between Harrell C-index estimates derived from the same patients. ΔC was calculated as C-index$_{HARP}$ − C-index$_{comparator}$, with positive values favoring HARP. Optimism-corrected C-indices were estimated separately using bootstrap internal validation with 1000 resamples...

Abbreviations: Hb, hemoglobin; AC, albumin-to-C-reactive protein ratio; HARP, hemoglobin–albumin–C-reactive protein index; CI, confidence interval.
